# Leaching of metal(loid)s from ashes of spent sorbent and stabilisation effect of calcium-rich additives

**DOI:** 10.1007/s11356-020-09269-z

**Published:** 2020-05-21

**Authors:** Alfreda Kasiuliene, Ivan Carabante, Hamid Sefidari, Marcus Öhman, Prosun Bhattacharya, Jurate Kumpiene

**Affiliations:** 1 Department of Civil, Environmental and Natural Resources Engineering, Luleå University of Technology, SE-97187 Luleå, Sweden; 2 Department of Engineering Sciences and Mathematics, Luleå University of Technology, SE-97187 Luleå, Sweden; 3 Department of Sustainable Development, Environmental Science and Engineering, KTH Royal Institute of Technology, Teknikringen 10B, SE-100 44 Stockholm, Sweden

**Keywords:** Arsenic, Heavy metals, Lime, Incineration, Iron-coated peat, Thermochemical equilibrium calculations

## Abstract

Contaminated water with multiple contaminants, including As, Cr, Cu and Zn, was treated with a sorbent prepared by coating peat with Fe oxides. Because As has a relatively little explored market, the regeneration of the spent sorbent was not feasible. Meanwhile, the disposal of As wastes in landfills can cause landfill leachate treatment problems. Under the reducing conditions prevailing at landfills, As(V) is reduced to As(III), which is a toxic and more mobile form. In this study, incineration was explored as a management option to treat the spent sorbent that was loaded with As, Cr, Cu and Zn. The first objective of this study was to evaluate the leaching of these metal(loid)s from the ashes and compare it with the leaching from the spent sorbents before incineration. The second objective was to evaluate the leaching behaviour when the spent sorbent was co-incinerated with a Ca-rich additive (lime). To achieve these objectives, the obtained ashes were subjected to leaching tests, sequential extraction, and X-ray diffraction analyses. After the incineration, the ash content ranged from 9 to 19% of the initial mass of the spent sorbents. The leaching of As, Cu and Zn decreased compared with that from the spent sorbents before the thermal treatment because of the high incineration temperatures and/or co-incineration with lime. However, the leaching of Cr increased, which would hinder the disposal of the obtained ashes in a landfill because the limit value for disposal at a landfill for hazardous wastes was exceeded by 50 times. However, co-incineration with 10 wt% lime significantly decreased the leaching of Cr as a result of the formation of water-insoluble Ca-Cr compounds.

**Electronic supplementary material:**

The online version of this article (10.1007/s11356-020-09269-z) contains supplementary material, which is available to authorized users.

## Introduction

Naturally metal(loid)s are found in the Earth’s crust, and they are dispersed in the environment by weathering. Usually, soils contain a full range of metal(loid)s, but because geochemical cycles are very slow, metal(loid)s are present at trace concentrations. Some metal(loid)s (i.e. micronutrients, such as Cr, Cu and Zn) are essential to living organisms in small concentrations, but higher doses can easily become toxic (Kabata-Pendias 2011). Nonessential metal(loid)s (e.g. As, Cd, Hg and Pb) become toxic as soon as they enter a living organism. Metal(loid)s then interact with biomolecules, disrupt essential biological functions and cause negative effects (Gall et al. 2015). Through bioaccumulation and biomagnification, metal(loid)s are transferred from terrestrial and aquatic ecosystems into the food chain and pose risks to human health (Alexander and Fairbridge 1999).

Increased As concentrations in groundwater can occur via natural and anthropogenic means. The natural sources are mainly geogenic. The anthropogenic ones include mining and smelting of As-rich materials, use of As-containing pesticides, wood preservation and glasswork. Metals like Cr, Cu and Zn are often found at elevated concentrations as co-contaminants (Bhattacharya et al. 2002a, b; Townsend et al. 2004). Various biological/biochemical, chemical and physico-chemical technologies are applied to treat contaminated groundwater to prevent the infiltration of contaminants into deep-lying aquifers (Hashim et al. 2011; Mudhoo et al. 2012; Ahmad et al. 2017; Azimi et al. 2017).

Adsorption is one of the well-established conventional methods used to remove metal(loid)s from contaminated water. A few examples of sorbents with high adsorption capacities are activated alumina, granular ferric hydroxide, Fe oxide-coated sand, activated carbon, clay minerals and zeolites (Sarkar and Paul 2016; Carolin et al. 2017; Uddin 2017). However, because of the different chemical characteristics of the contaminants, multi-element contaminated water usually requires more than one treatment to reduce the risks caused by all of the present contaminants. This has two important implications. First, it leads to the generation of spent sorbents loaded with metal(loid)s. Given the increased health concerns related to the presence of As and metals in drinking water, as well as the enactment of more stringent regulations, it can be expected that even higher amounts of spent sorbents will be generated in the future. Today, there is a lack of appropriate management and disposal methods for waste loaded with metal(loid)s, and with As in particular. Second, the production of highly efficient commercial sorbents such as silica gel or alumina often requires the use of virgin materials, which can be costly and unsustainable from the environmental point of view.

To tackle these issues, we combined two highly efficient sorbents, namely peat and Fe oxide, into one sorbent (iron–peat) that can simultaneously remove cationic (Cu, Zn) and anionic (As (arsenate, arsenite), Cr (chromate, dichromate)) contaminants from contaminated water (Kasiuliene et al. 2018; Kasiuliene et al. 2019a). Thus, the amount of the resulting spent sorbent is lower in comparison with the case where several sorbents are used. Furthermore, the peat and Fe oxides were both waste-based materials (by-products), which already needed management. Therefore, the return of these materials back to society could have a positive effect from the circular economy point of view, as well as being cost-effective. The necessity of coating peat with Fe oxides in order to achieve the simultaneous removal of several contaminants was confirmed using only non-coated peat or only Fe oxides (coated on sand), in which case each was effective only for certain elements, but not for all of the investigated elements (As, Cr, Cu and Zn) simultaneously. The efficiency of the iron–peat was attributed to the increased Fe content, larger specific surface area and the presence of organic matter (Kasiuliene et al. 2019a).

After a sorbent is exhausted and it is not possible/feasible to regenerate/recycle it, it becomes waste and requires management. A common practice to dispose of waste loaded with As is landfilling because As has a relatively little explored market, and its regeneration is expensive. However, when oxidised wastes loaded with As are exposed to a reducing environment such as that of a landfill, the release of As into the landfill leachate can drastically increase because of the reductive dissolution of Fe oxides and microbial activity (Ghosh et al. 2004; Kumpiene et al. 2009; Clancy et al. 2013). In the case of iron–peat, the spent sorbent could not be disposed of at landfills for hazardous waste because the leaching of As and Cr exceeded the limit values (Council Decision 2003/33/EC). Furthermore, when the leaching of metal(loid)s was tested under the reducing conditions, the leaching of As was substantially higher (up to 40%) than that under oxidising conditions. It was also determined that about one-third of the As(IV) was reduced to As(III). Although the leaching of Cu and Zn was less affected by the reducing environment, there was an indication that from the long-term perspective, elevated concentrations of these metals could be expected in the landfill leachate along with As (Kasiuliene et al. 2019a).

Because peat has a relatively high calorific value, thermal treatment could be a viable option to treat peat-based spent sorbents. In general, the thermal treatment of waste plays a key role in modern waste management systems. It is a preferred alternative in solid waste management because landfilling is becoming more difficult as a result of high costs, diminishing land availability and stricter regulations (Veli et al. 2008). The main thermal treatment methods include incineration, gasification and pyrolysis, where energy is produced in the form of heat, power and syngas. The incineration of waste offers several advantages over traditional landfilling, such as hygienisation, the destruction of organic pollutants and the reduction of the volume and mass of the solid waste (Lundholm et al. 2007). In the case of incinerating our spent sorbent, As could be concentrated in a relatively small body of ash and disposed of at a landfill (as well as removed from society). However, landfilling ashes that contain high amounts of potentially leachable elements, without any pre-treatment, could still pose environmental risks.

Immobilisation is one of the conventional methods used to treat metal(loid)-contaminated soils, and among other immobilising agents such as P compounds and Fe or Mn oxides, materials rich in Ca are also being used (Bolan et al. 2014). As reported by Travar et al. (2015), the formation of poorly soluble Ca-As minerals such as calcium arsenate, weilite and jahnbaumite were responsible for the immobilisation of As in the contaminated soil, where a Ca-rich waste product derived from the air pollution controller in an incinerator was added. In the same study, it was reported that the addition of Ca had a slight mobilising effect on Cr and Cu. Lundholm et al. (2007) investigated the potential to stabilise As, Cr and Cu while co-incinerating CCA-wood mixed with peat, which had a high content of Ca and Al. It was reported that As and Cr formed refractory phases with Ca: Ca_3_(AsO_4_)_2_ and CaCrO_3_, CaCr_2_O_4,_ respectively. In the case of Cu, stable forms were obtained because of the increased Al content, e.g. CuAl_2_O_4_. The overall conclusion was that the addition of Ca-rich peat could reduce the volatilisation of As and Cr during incineration (Lundholm et al. 2007).

The first objective of this study was to evaluate the leaching of As, Cr, Cu and Zn from the resulting ashes and compare it with the leaching from the spent sorbents before the incineration. The second objective was to evaluate the leaching of the same metal(loid)s when the spent sorbent was co-incinerated with a Ca-rich additive. To achieve these objectives, the obtained ashes were subjected to leaching tests, sequential extraction and X-ray diffraction (XRD) analyses. Thermochemical equilibrium calculations (TECs) were used to predict the co-existing phases for the different experimentally tested scenarios to help interpret the experimental findings.

## Materials and methods

### Spent sorbents

Heat-treated peat was obtained from Geogen Produktion AB, Sweden. This company produces heat-treated peat granulate as an environmentally compatible oil adsorption agent, and particles smaller than 2 mm are discarded during its production. This heat-treated peat residue, including (i) uncoated peat and (ii) peat coated with ferric ferrous hydrosol (Rekin, Lithuania), was used for the simultaneous adsorption of As, Cr, Cu and Zn from a contaminated solution. Details about the sorbent preparation and adsorption experiment can be found in Kasiuliene et al. (2019b). Briefly, the sorbents were mixed with a metal(loid) solution at a liquid-to-solid (L/S) ratio of four and then dried out at room temperature. A contaminated solution containing 1 g L^−1^ of As and 4 g L^−1^ of Cr, Cu and Zn was prepared by dissolving analytical grade chemicals, namely NaH_2_AsO_4_ (Honeywell Riedel-de Haen AG, 99%), K_2_Cr_2_O_7_ (VWR International, 99%), CuCl_2_·2H_2_O (Merck, 98%) and ZnCl_2_ (Merck, 98%), in a 0.1 M KNO_3_ solution (Merck, 99%). Hereafter, the spent iron–peat sorbent is referred to as ‘iron–peat’, and the spent peat sorbent is referred to as ‘peat’. The elemental compositions of the spent sorbents were determined using inductively coupled plasma optical emission spectrometry (ICP-OES) (Optima 8300, Perkin-Elmer) after wet digestion with *aqua regia* in a microwave oven (CEM Mars 5) at 190 °C. A batch leaching test (L/S = 10) was performed as described in the standard ‘Characterization of waste – Leaching Compliance test for leaching of granular waste materials and sludges’ (SS-EN 12457–4). The total metal(loid) concentrations in the spent sorbents and the leached out concentrations are presented in Table 1.

**Table 1:** Total metal(loid) concentrations (mg kg^−1^) in spent sorbents and leached out concentrations (mg kg^−1^) ± standard deviation of the mean, *n* = 3 (Kasiuliene et al. 2019b)

Sample	As	Cr	Cu	Fe	Zn
Peat, total	411 ± 53	3673 ± 215	3697 ± 76	20,133 ± 1593	3728 ± 92
Peat, leach	29.4 ± 0.5	8.9 ± 0.1	27.9 ± 0.6	3.4 ± 0.2	6.9 ± 0.4
Iron-peat, total	993 ± 50	3821 ± 114	3795 ± 58	63,295 ± 2659	2823 ± 69
Iron-peat, leach	0.53 ± 0.16	11.6 ± 0.2	39.9 ± 0.5	38.5 ± 0.3	272 ± 1

### Addition of lime

A liming by-product composed mainly of Ca-carbonate (CaCO_3_) and Ca-hydroxide (Ca(OH)_2_) was added to the iron–peat prior to the incineration experiment. This by-product is derived from the production of pulp for the paper industry (Mewab, Sweden). The lime was dried at 105 °C and crushed with a mortar to obtain a homogenous powder (particle size < 0.1 mm). Then, based on the dry weight of the iron–peat (Kasiuliene et al. 2019b), lime at 10 wt% was mixed into the spent iron–peat. Hereafter, the mixture of iron–peat and lime is referred to as ‘IP–lime’. The elemental composition analyses and leaching tests were performed with the IP–lime following the same procedures as for the spent sorbents. The pH of the lime was measured in a water suspension at a 1v:1v ratio.

### Incineration

The spent sorbents were incinerated in a high-temperature furnace (Entech Energiteknik AB, Sweden) at the following two temperatures of interest: 850 °C and 1100 °C in stagnant air. Such temperatures are defined in the Directive on the incineration of waste (Directive 2000/76/EC) for non-hazardous and hazardous waste, respectively. The volume (V) of the furnace was 7.8 dm^3^. Each sample was carefully weighed to reach 2.50 ± 0.01 g of TS and placed into alumina crucibles (*V* = 0.4 cm^3^). Then, the oven was heated to the respective target temperatures at a heating rate of 10 °C min^−1^, and the residence time was 0.5 h. After cooling, the ash content was determined gravimetrically. The ashes were kept in glass jars for further analyses. The ashes obtained after the incineration of the spent peat, iron–peat and IP–lime at 850 °C are referred to as ‘peat 850’, iron–peat 850’ and ‘IP–lime 850’, respectively. The ashes obtained after the incineration at 1100 °C are referred to as ‘peat 1100’, iron–peat 1100’ and ‘IP–lime 1100’, respectively.

### Thermal properties of spent sorbents

Prior to incineration, the calorific values of the spent sorbents, including the mixture with lime, were determined using a combustion calorimeter (IKA C 200).

An evolved gas analysis was performed during the thermogravimetric (TG) analysis. It was carried out in a NETZSCH thermal analysis STA 409 instrument with simultaneous TG analysis with a sensitivity of ± 1 μg (TGA), coupled with a differential thermal analysis (DTA). The analyses were performed using alumina crucibles under a synthetic air atmosphere. Each sample was heated from room temperature to 1100 °C at a heating rate of 10 °C min^−1^. Thereafter, an isothermal stage was maintained for 20 min. A constant flow rate of 200 ml min^−1^ of synthetic air was used during the analyses.

### Determination of metal(loid) behaviour in ashes

Metal(loid) leaching from the ashes was determined after the batch leaching test at L/S = 10 (SS-EN 12457-4). The obtained values were compared with the leaching limit values applicable to the acceptance of waste at landfills (Council Decision 2003/33/EC).

The total metal(loid) concentrations were determined following a four-stage acid-extraction procedure at an accredited laboratory (ALS Scandinavia, Sweden).

XRD analyses were performed at the Helmholtz Institute Freiberg, Germany. The ash samples were wet-milled with ethanol to reach a grain size of approximately 4 μm. The measurements were done with a PANalytical Empyrean diffractometer (Malvern Panalytical, Kassel, Germany) equipped with a co-tube (λ = 1.789 Å), an Fe filter, an automatic divergence slit to provide a constant irradiated area on the sample (12 × 15 mm^2^) and a PIXcel 3Dmedipix area detector. Samples were measured in the 2θ range of 5–80°. The system was operated at 35 kV and 35 mA. The NIST 660 °C standard was prepared and measured in the same manner (except for the milling). The HighScore Plus software and ICDD (International Centre of Diffraction Data) PDF-4 (2019) database were used for the qualitative phase analysis.

A sequential extraction procedure adopted from Tessier et al. (1979), where the first step was modified after Bódog et al. (1996), was applied to the spent sorbents and ashes. Briefly, the exchangeable fraction (I) was obtained after extraction for 16 h with a 1.0 M ammonium acetate (VWR International, 98.6%) solution at pH 6.5; the acid-soluble fraction (II) was obtained after extraction for 5 h with a 1.0 M sodium acetate (Merck, 99%) solution at pH 5.0; the Fe-Mn oxide fraction (III) was obtained after a 6 h extraction with a hydroxyl-ammonium chloride (Merck, 99%) solution at pH 2 in a heated water bath at 96 °C and the oxidisable fraction (IV) was obtained after 1 h of extraction with hydrogen peroxide (Merck, 35%) in a heated water bath at 85 °C. The sequential extractions were completed by extracting the residual fraction (V) with *aqua regia* at 190 °C for 10 min. The extractions were performed in triplicate, and the extracts were filtered through 0.45 μm nitrocellulose filters, acidified (except the residual fraction) and analysed with ICP-OES.

### TECs

A global thermodynamic equilibrium modelling approach was used to predict the co-existing phases for the different experimentally tested scenarios to help interpret the experimental findings by employing the Gibbs free energy minimisation approach where it was assumed that (i) all elements are homogenously distributed in the media and (ii) they are in equilibrium. The TECs were performed using the FToxid and FactPS databases (Bale et al. 2009; Bale et al. 2016). Solid solution phases were iteratively selected from the FToxid database. For the mass balance calculations, the total metal(loid) concentrations of peat, iron–peat and IP–lime were used as inputs. The elements included in the TECs were Si, Al, Ca, Fe, K, As, Cr, Cu and Zn. The ultimate (elemental) analysis of the spent peat (C, H, N, O, S, Cl) (Kasiuliene et al. 2019b) was used to generate the typical gaseous atmosphere present at incineration facilities. An air factor of 1.5 (50% excess air) was assumed as sufficient to simulate the oxidising gaseous atmosphere. The equilibrium partial pressures were used for H_2_O and all the other gas species, i.e. the partial pressures were not constrained. In addition, complementary N_2_ and argon (as balance) were used. The partial pressure of O_2_ varied from the assumed 0.06 atm up to 0.21 atm in air. However, no significant difference was observed in the predicted phases and the distribution of the trace elements of interest.

## Results and discussion

### Waste mass reduction

**Fig. 1:**
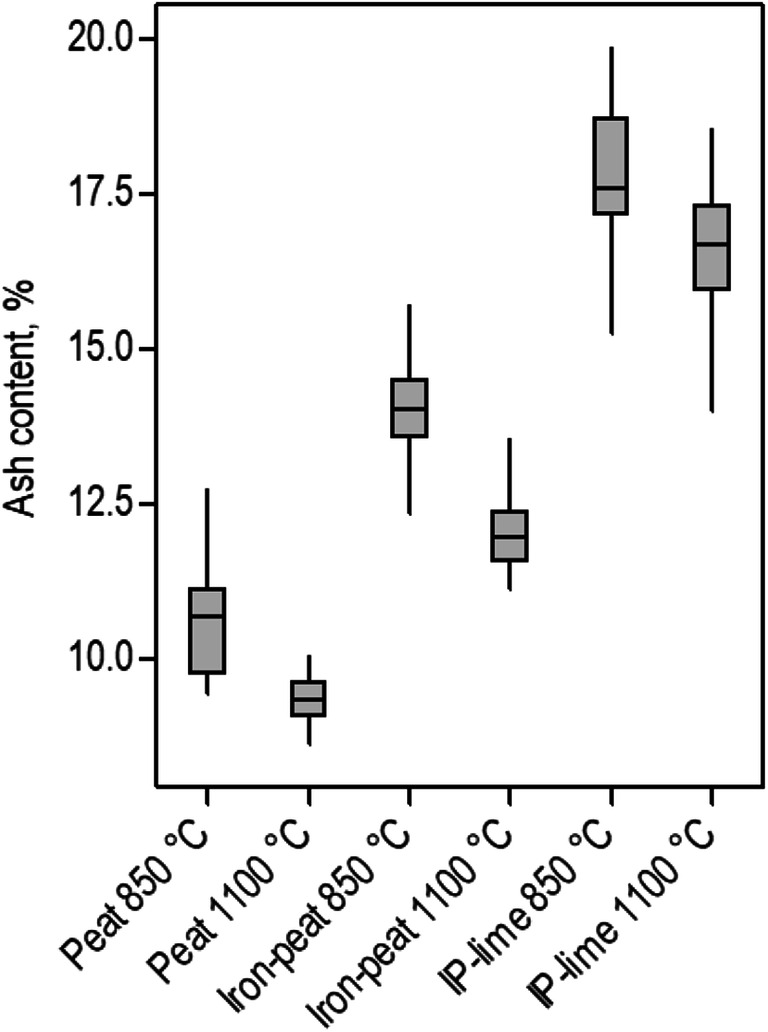
Ash contents of the incinerated spent sorbents. Error bars represent standard deviation of the mean, *n* = 14

The ash contents after incinerating the peat, iron–peat and IP–lime at 850 °C and 1100 °C are presented in Fig. 1. Table 2 lists the average total concentrations of As, Cr, Cu, Fe and Zn in the ashes. Incinerating the spent sorbents at higher temperatures resulted in lower ash contents. Thus, higher contents of As, Cr, Cu and Zn were concentrated in a smaller waste body. The obtained ash contents ranged from approximately 9 –19 wt%. The addition of inorganic compounds (Fe oxides and lime) decreased the calorific value of the spent sorbents (i.e. fuel) and increased the ash content. Peat without any modifications had a calorific value of 19.7 ± 1.2 MJ kg^−1^, which decreased to 18.8 ± 0.8 MJ kg^−1^ when coated with Fe oxides and decreased even further to 17.31 ± 2.1 MJ kg^−1^ when lime was added.

**Table 2:** Total metal(loid) concentrations (g kg^−1^) in ashes ± standard deviation of the mean, *n* = 3

Sample	As	Cr	Cu	Fe	Zn
Peat 850	4.9 ± 0.3	29.9 ± 1.0	34.3 ± 2.1	115 ± 5	24.3 ± 0.8
Peat 1100	5.9 ± 0.2	35.3 ± 0.9	35.9 ± 0.7	91 ± 3	25.3 ± 0.6
Iron-peat 850	5.6 ± 0.3	23.4 ± 1.1	25.1 ± 1.1	109 ± 1	15.9 ± 0.7
Iron-peat 1100	6.2 ± 0.2	25.4 ± 0.5	26.3 ± 0.1	93 ± 1	17.9 ± 0.7
IP-lime 850	3.1 ± 0.1	17.5 ± 1.6	21.3 ± 1.2	122 ± 9	14.7 ± 1.2
IP-lime 1100	3.8 ± 0.2	18.6 ± 1.5	18.9 ± 0.8	132 ± 7	14.4 ± 0.9

The determined calorific values of the spent sorbents were within the range of calorific values typical for peat (13.6–25.4 MJ kg^−1^) (Lehtovaara and Salonen 2012). Although in this study the incineration of spent sorbents was considered to be a way to reduce the mass (and volume) of waste, waste-to-energy technology should also be explored because, in general, it not only provides renewable sources of energy but also has the potential for recycling solid wastes (preferably with a high organic content) (Kothari et al. 2010). In this study, the peat and ferric ferrous hydrosol (for coating the peat) were both waste-based materials. In addition, the lime used to reduce the leaching of contaminants from the ashes was also an industrial residue. Therefore, this process of utilising waste-based materials to clean out contaminated water, followed by the co-incineration of several waste materials at the same time, is advantageous from the circular economy and environmental points of view.

### Metal(loid) leaching from ashes

A previous study (e.g. Lundholm et al. 2007) reported that the incineration of As-containing wastes was not desirable because of the low volatilisation temperature of As. The volatilisation of pure As_2_O_5_ can start at approximately 600 °C, whereas a much lower volatilisation temperature was reported (320 °C) when a mixture of As_2_O_5_ and sawdust started to smoulder. In addition, Zn also belongs to a group of semi-volatile elements, which are usually depleted in the bottom ash and enriched in the fly ash (Lundholm et al. 2007). In this study, during the evolved gas analyses, volatile substances containing metal(loid)s did not evolve during the combustion of the sample while performing TGA. It is likely that the metal(loid) content present in the spent sorbents was too low for substantial metal(loid)-gas formation. Therefore, it was assumed that the entire content of As, Cr, Cu and Zn was present merely in the ashes. However, it was not possible to explain what caused the Fe losses during the incineration of the peat and iron–peat at 1100 °C; the concentration was higher in the ashes obtained at 850 °C.

**Fig. 2:**
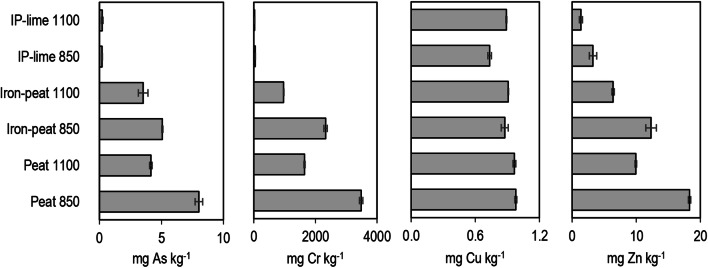
Average metal(loid) concentrations in the ash leachates. Error bars represent standard deviation of the mean, *n* = 3

Figure 2 presents the As, Cr, Cu and Zn concentrations in the leachates from the ashes after the standardised batch leaching test at L/S = 10. The abovementioned test is also a compliance test, which can be used to confirm the appropriate type of landfill for the disposal of the waste. Based on the contaminant concentrations detected in the leachate, the waste can be deposited at landfills for (i) inert, (ii) non-hazardous or (iii) hazardous wastes. In previous studies (Kasiuliene et al. 2019a, b), it was concluded that the spent peat and iron–peat could not be landfilled at landfills for hazardous waste. The leaching of As, which intensified drastically under the reducing conditions, was one of the main factors hindering the landfilling.

The leaching limit values for waste that can be accepted at landfills for hazardous waste are as follows: 25 mg kg^−1^ of As, 70 mg kg^−1^ of Cr, 100 mg kg^−1^ of Cu and 200 mg kg^−1^ of Zn (Council Decision 2003/33/EC).

As shown in Fig. 2, the leaching of As from the ashes in all cases was below the limit value. Incinerating the spent sorbents at the higher temperature (1100 °C) slightly reduced the leaching of As from the ashes, whereas the addition of lime had a substantially higher effect. The leaching of As from the IP–lime ashes was 30 times lower than the leaching from the iron–peat ashes.

The concentration of Cu in the leachates was approximately 1 mg kg^−1^, which corresponded to 1% of the limit value. The incineration of the spent sorbents at different temperatures, as well as the addition of lime, did not have significant effects on the leaching of Cu.

The leaching of Zn was also below the limit value. However, because Zn is usually more water-soluble than Cu (Kabata-Pendias 2011), incinerating the spent sorbents at the higher temperature (1100 C°) reduced the leaching of Zn from the ashes by approximately two times compared with the leaching from the ashes obtained at 850 °C. The co-incineration with lime reduced the leaching of Zn from the IP–lime ashes by three times compared with the iron–peat ashes.

Among the analysed metal(loid)s, Cr exhibited the most intensive leaching from the ashes of the spent sorbents. The Cr concentration in the leachate from the peat 850 ashes was almost 50 times above the limit value. Even though the leaching of Cr from the ashes was significantly reduced at the higher incineration temperature (1100 °C), it still exceeded the limit value by several times. Under the given experimental conditions, the leaching of Cr decreased below the leaching limit value when the spent sorbent was co-incinerated with lime.

In all cases, the addition of lime, together with the higher incineration temperature, had a positive synergistic effect on the metal(loid) stability in the ashes. Under the given experimental conditions, the co-incineration of the spent sorbents with the 10 wt% lime additive increased the ash content by 6–7% compared with the ash content before the lime addition (Fig. 2). Therefore, the increased utilisation of lime should be undertaken with caution because it might result in high ash loads from the use of inorganic lime. Additionally, facilities for incinerating hazardous waste operating at 1100 °C are less common in Sweden and the rest of Europe. Therefore, the additional transportation costs would increase the overall treatment costs and would have a negative effect on the environment. For this reason, a compromise between the slightly increased incineration efficiency, treatment costs and impact on the environment needs to be carefully considered.

### Metal(loid) distribution in ashes

**Fig. 3:**
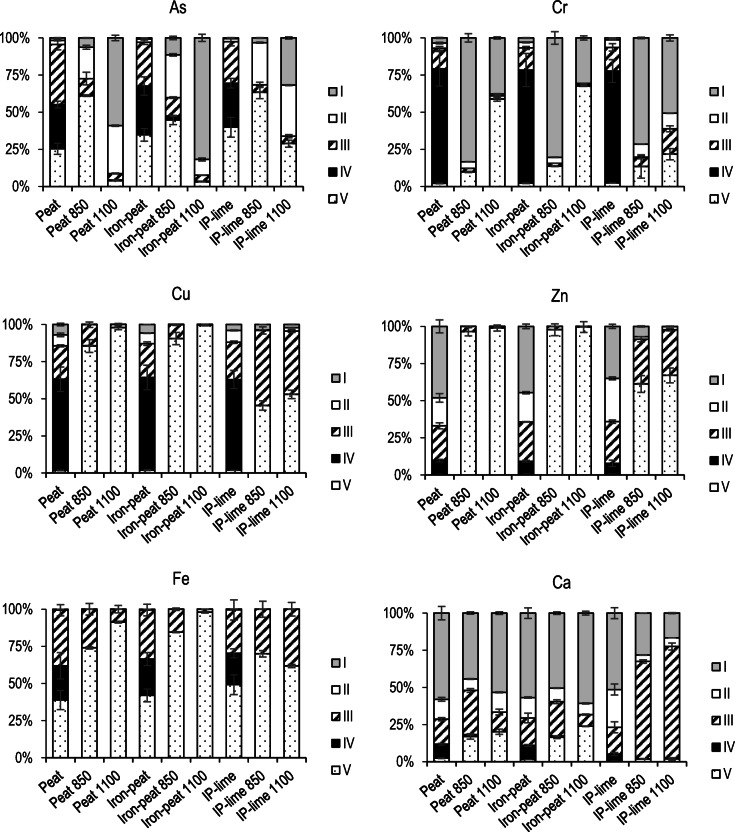
Fractionation of As, Cr, Cu, Zn, Fe and Ca in the spent sorbents and in ashes obtained at 850 °C and 1100 °C. I, exchangeable; II, acid-soluble; III, Fe-Mn oxide; IV, oxidisable; V, residual fraction. Error bars represent standard deviation of the mean, *n* = 3

Figure 3 presents the different metal(loid) distribution fractions found in the ashes. It was anticipated that the fractionation of the As, Cr, Cu and Zn would be in line with the results from the standardised batch leaching. However, while this was true for Cr, Cu and Zn, it was not true for As. The main trend for Cr and Zn was that in the ashes obtained at the higher temperature (1100 °C), the exchangeable fraction was smaller, while the residual fraction was larger compared with that in the ashes obtained at the lower temperature (850 °C). The decrease in the exchangeable (water-soluble) fraction explains why Cr and Zn leached out less from the ashes obtained at 1100 °C during the standardised batch leaching test. The exchangeable fraction of Cu in all cases corresponded to less than 5%. Thus, the leaching of Cu during the batch leaching test was negligible. In contrast to Cr and Zn, the fractionation of As contradicted the results of the batch leaching test. The ashes obtained at the lower incineration temperature (850 °C) in all cases had a smaller exchangeable fraction, whereas the residual fraction was always larger compared with the ashes obtained at 1100 °C. However, in the standardised batch leaching test, significantly less As leached out from the ashes obtained at 1100 °C. At temperatures below 650 °C, the TECs (Supplementary Figs. 4,5 and 6) predicted that the As would be found in complexes with Ca, whereas K-As complexes would be dominant with increasing temperature. However, the sequential extraction analysis indicated that As was associated with Ca after the incineration of the spent peat and iron–peat at 850 °C. Upon increasing the temperature (to 1100 °C), K-As complexes were predicted (Supplementary Figs. 4, 5 and 6). Because Ca-As is less soluble than K-As (Rochette et al. 1998), a smaller fraction of the exchangeable As and larger fraction of the residual As was found in the ashes obtained at lower temperatures. This is in line with the fractionation of Ca determined by the sequential extraction (Fig. 3). The ashes obtained at the higher temperature (1100 °C) had a slightly larger fraction of the exchangeable Ca and a larger residual fraction. In addition, the Fe-Mn oxide fraction of Ca was also larger in the ashes obtained at 850 °C compared with that in the ashes obtained at 1100 °C. However, the presence of Ca-As was not detected by the XRD analysis, most likely because the Ca-As appeared to be in an amorphous phase rather than crystalline. According to the phase fitting calculations for the XRD patterns, a 16–31 wt% corresponded to amorphous phase for the peat and iron–peat ashes. The amorphous phase was slightly lower for the ashes obtained at the higher temperature.

In Kasiuliene et al. (2018), it was reported that in the spent iron–peat sorbent, As was adsorbed mainly onto ferrihydrite. However, in this study, the XRD analysis showed that haematite was the dominant crystalline Fe-bearing mineral in the ashes (Supplementary Figs. 8, 9, 10 and 11). Therefore, it is likely that upon the Fe oxide transformation from ferrihydrite to haematite due to the high-temperature oxidising atmosphere, a share of As was disassociated from the haematite and, together with Ca, went to the amorphous phase. It was shown in Zhu et al. (2006) that the solubility of Ca-As is pH dependant, with the least soluble compounds forming at pH values ranging between 11 and 13. In addition, it was shown that Ca-As compounds that precipitated under a low pH (3–5) had a more crystalline structure compared with Ca-As compounds obtained at a higher pH. This is in line with our study because it was not possible to detect crystalline Ca-As structures during the XRD analysis. Furthermore, because the solubility of Ca-As is low under high pH values, it explains the low leaching of As during the standardised batch leaching test, because the pH values of the leachates for all the samples ranged between 10 and 11. The association of As with either K or Ca was compatible with the increased As extraction in the exchangeable fraction, ammonium acetate solution under a much lower pH environment (6.5).

In the IP–lime ashes, Ca was mainly found in the Fe-Mn fraction. The TECs indicated a gradual increase in the Ca_2_Fe_2_O_5_ formation with an increase in the CaO content in the system (Supplementary Fig. 7). This was confirmed by the XRD results because Ca_2_Fe_2_O_5_ corresponded to 42–55% of the IP–lime ashes in the diffractogram (Supplementary Figs. 12, 13). It is very likely that the Ca incorporation into the Fe oxide structure altered the interaction between As and Fe, promoting the mobilisation of As. This was confirmed by the sequential extractions (Fig. 3), where As, which was found in the Fe-Mn oxide fraction (III) present in the IP–lime prior to incineration, disappeared from the IP–lime ashes. Instead, the exchangeable fraction of As increased. On the other hand, the amorphous phase in the IP–lime ashes increased (up to 43%) compared with that in the iron–peat ashes, which was poorly water-soluble during the standardised batch leaching test at the pH value of approximately 12.

The sequential extractions (Fig. 3) revealed that the Cr in the spent sorbents was mostly bound to the organic fraction, which became oxidised during the incineration; thus, a significant fraction of Cr became water-soluble. The TECs showed that water-soluble K-Cr oxides were dominant at temperatures below 800–900 °C (Supplementary Figs. 4, 5 and 6). As the temperature continued increasing, the melt fraction associated with Cr also increased, as well as the formation of corundum (Supplementary Fig. 7). For this reason, Cr leached less from the ashes obtained at the higher temperature (1100 °C). Because of the increased CaO content, corundum was no longer detectable in the IP–lime ashes. Instead, the TECs predicted that Cr would mostly be found in the form of the water-insoluble CaCr_2_O_4_ spinel (Supplementary Figs. 4, 5 and 6), which was in line with the substantially reduced leaching of Cr during the standardised batch leaching test.

The sequential extraction showed (Fig. 3) that in the peat and iron–peat ashes, irrespective of the incineration temperature, almost all of the Zn was found in the residual fraction. However, more substantial differences were observed in the Zn leaching from the ashes during the leaching test. Zinc leached more from the ashes obtained at the lower temperature (850 °C), while the exchangeable fraction of Zn during the sequential extractions was very small. It is likely that the pH of less than six that was prevalent during the sequential extractions hindered the extraction of the I–IV fractions of Zn. During the batch leaching test, a higher pH (around 11) resulted in a higher mobility for Zn. The co-incineration with lime decreased the leaching of Zn from the IP–lime ashes because of the predicted formation of (i) slag and (ii) spinel compounds with Fe (Supplementary Figs. 4, 5 and 6).

The behaviour of Cu was very similar to that of Zn. As predicted by the TECs, the formation of slag and spinel (Supplementary Figs. 4, 5 and 6) could potentially be responsible for the stabilisation of Cu in the ashes and its very weak leaching during the standardised batch leaching test.

In summary, As was associated with ferrihydrite before the incineration. Then, as it was transformed into haematite with the increasing temperatures, As became associated with Ca in the poorly water-soluble amorphous phase, which then explained the low leaching of As during the standardised batch leaching test. The formation of the water-insoluble spinel of CaCr_2_O_4_ resulted in the decreased leaching of Cr when the iron–peat was co-incinerated with lime. In the case of Cu and Zn, the formation of slag and spinel resulted in weak leaching from the ashes.

## Conclusions

In this study leaching of metal(loid)s was used as one of the main indicators determining whether ashes of spent sorbent could be landfilled or not. Need for the treatment occurred because leaching of As from the spent sorbent (prior incineration) was exceeding the limit values. The leaching of As from the IP–lime ashes was 30 times lower than the leaching from the iron–peat ashes.

The leaching of Cr from the ashes was significantly higher than that from the spent sorbents and exceeded the limit values by 50 times. The increased leaching occurred because in the spent sorbents, a significant fraction of Cr was associated with an oxidisable fraction, which was then followed by an immense transformation into exchangeable Cr during the incineration. The addition of a Ca-rich lime additive decreased the leaching of all the investigated metal(loid)s, but the highest effect was observed in the case of Cr. The predicted formation of the water-insoluble spinel of CaCr_2_O_4_ may have been responsible for the reduction of the Cr leaching below the limit value for waste acceptable at landfills for hazardous waste.

Although the leaching of Cu and Zn from the spent sorbents was already below the limits for waste acceptable at landfills for hazardous waste, the leached out concentrations decreased even further after the incineration.

Through incineration, it was possible to achieve a significant waste mass reduction. The ash content after the incineration was 9–19 wt%. Although the addition of lime decreased the metal(loid) leaching from the ashes, co-incineration with higher shares of lime needs to be considered with caution, because it would increase the ash content.

## Electronic supplementary material

ESM 1 (DOCX 836 kb)
