# Supplementary material for: Leaching of metal(loid)s from ashes of spent sorbent and stabilisation effect of calcium-rich additives
Source: Environ Sci Pollut Res Int. 2020 May 21;27(23):29248–56. doi: 10.1007/s11356-020-09269-z (PMC7376079; doi:10.1007/s11356-020-09269-z)
Supplement: Supplementary file 1 — (DOCX 836 kb) [file 11356_2020_9269_MOESM1_ESM.docx]

**
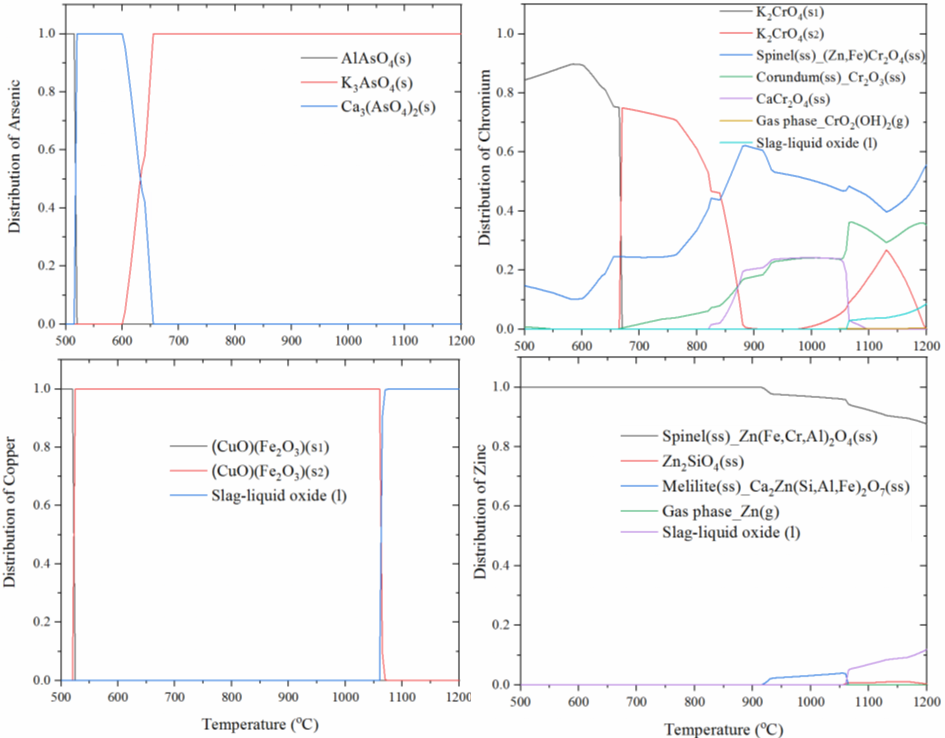
**

**Fig 4** Distributions of As (top left), Cr (top right), Cu (bottom left), and Zn (bottom right) in peat ashes as predicted by thermochemical equilibrium calculations (TECs) within the temperature range of 500–1200 °C


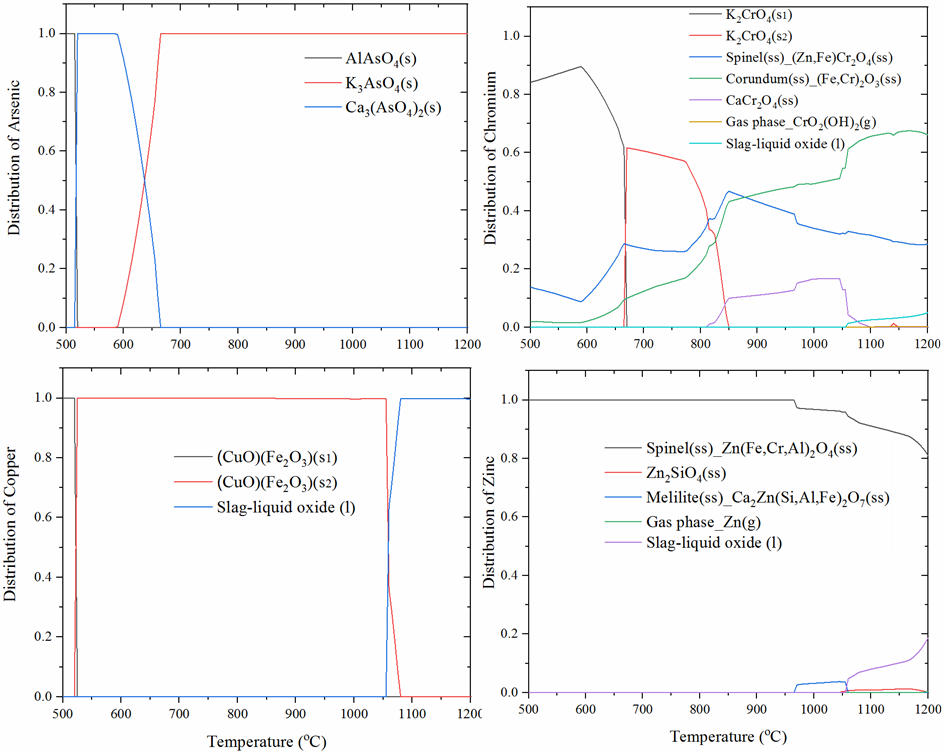


**Fig 5** Distributions of As (top left), Cr (top right), Cu (bottom left), and Zn (bottom right) in iron–peat ashes as predicted by thermochemical equilibrium calculations (TECs) within the temperature range of 500–1200 °C


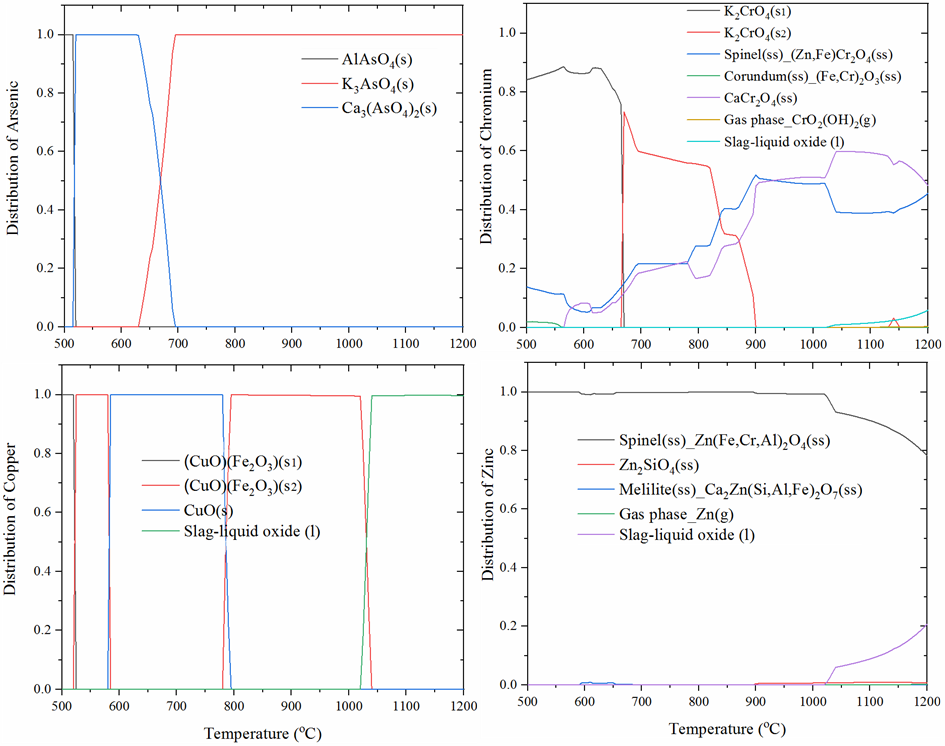


**Fig 6** Distributions of As (top left), Cr (top right), Cu (bottom left), and Zn (bottom right) in IP–lime ashes as predicted by thermochemical equilibrium calculations (TECs) within the temperature range of 500–1200 °C


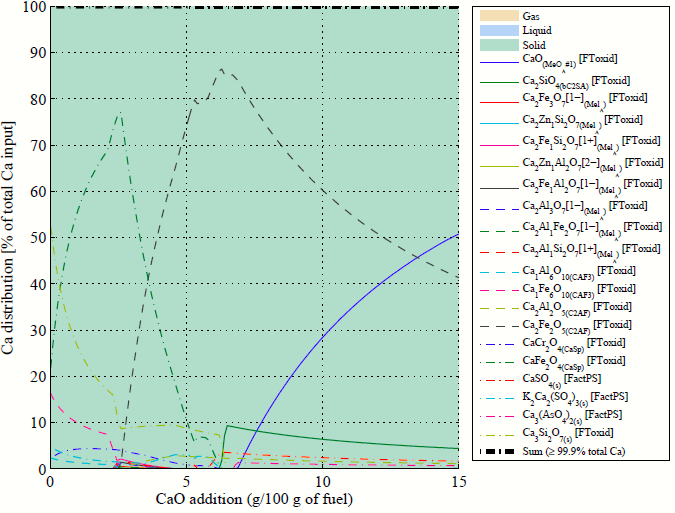


**Fig 7** Distribution of Ca with increasing addition of CaO to spent peat sorbent as predicted by thermochemical equilibrium calculations (TECs) at 850 °C

***
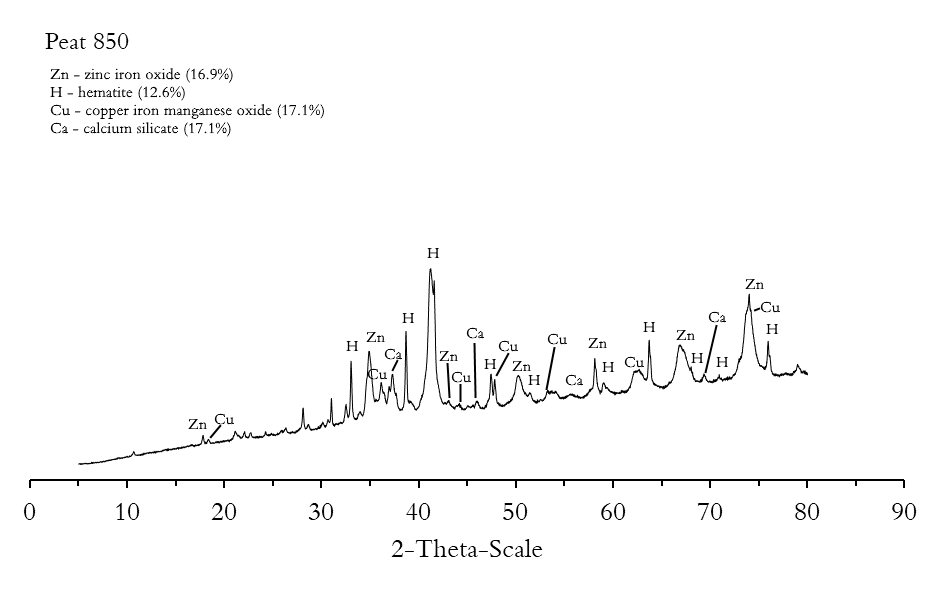
***

**Fig 8** XRD spectra of peat ashes obtained at 850 °C


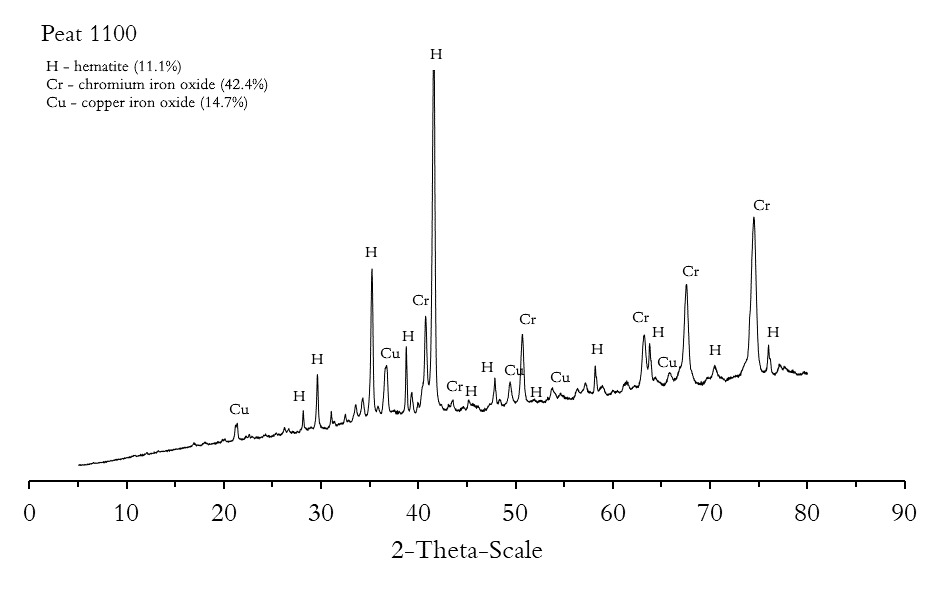


**Fig 9** XRD spectra of peat ashes obtained at 1100 °C


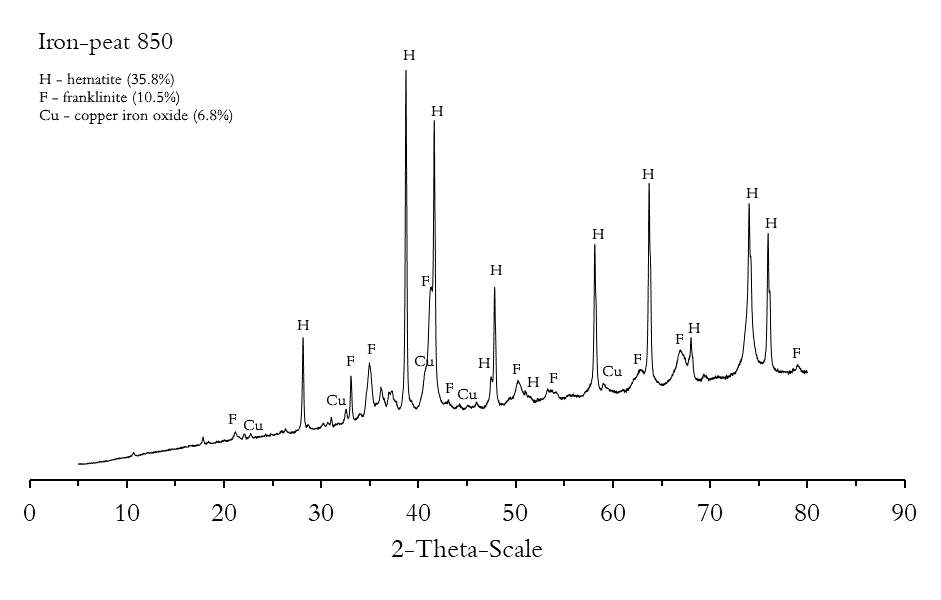


**Fig 10** XRD spectra of iron–peat ashes obtained at 850 °C


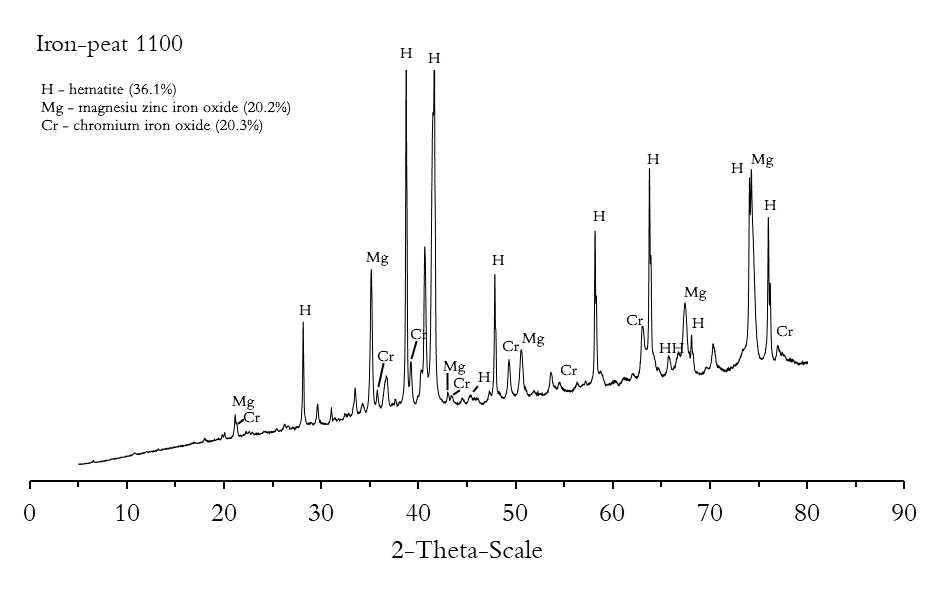


**Fig 11** XRD spectra of iron–peat ashes obtained at 1100 °C


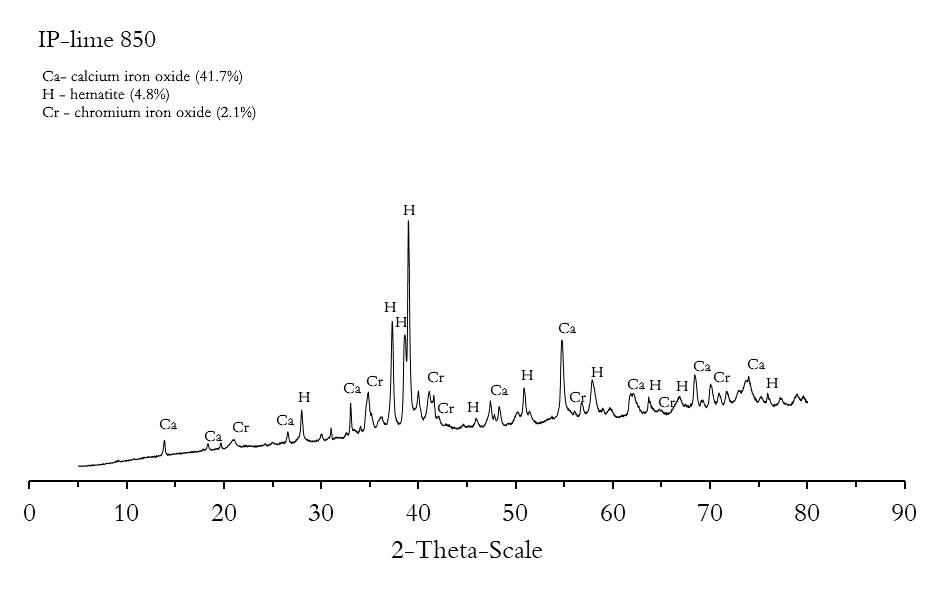


**Fig 12** XRD spectra of IP–lime ashes obtained at 850 °C


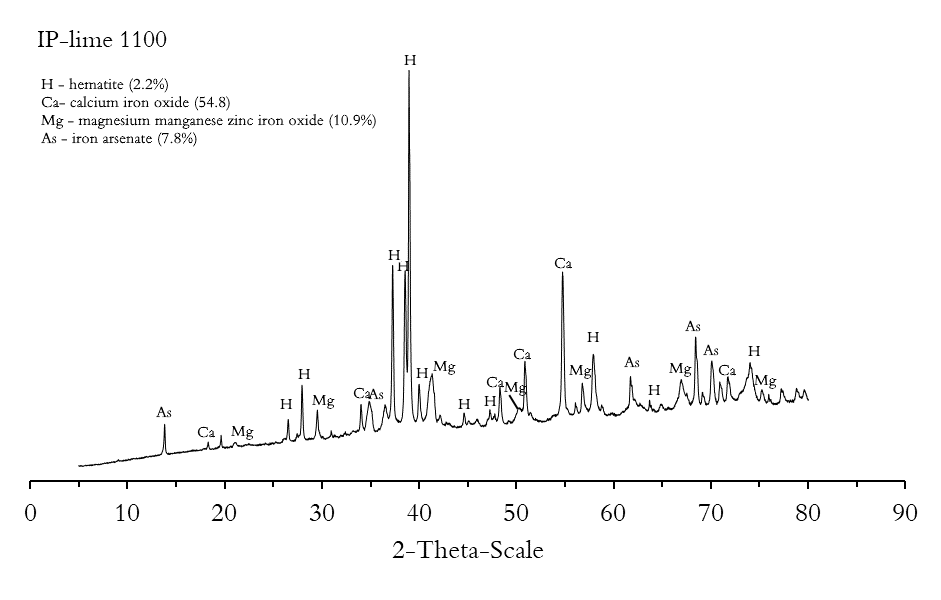


**Fig 13** XRD spectra of IP–lime ashes obtained at 1100 °C
